# Trends in null hypothesis significance testing: Still going strong

**DOI:** 10.1016/j.heliyon.2024.e40133

**Published:** 2024-11-06

**Authors:** Frank Emmert-Streib

**Affiliations:** Predictive Society and Data Analytics Lab, Faculty of Information Technology and Communication Sciences, Tampere University, Tampere, Finland

**Keywords:** Null hypothesis significance testing, Statistical inference, Change-point analysis, Trends

## Abstract

Null hypothesis significance testing (NHST) is among the most prominent and widely used methods for analyzing data. At the same time, NHST has been criticized since many years because of misuses and misconceptions that can be found extensively in the scholarly literature. Furthermore, in recent years, NHST has been identified as one reason for the replication crisis because many studies place too much emphasis on statistical significance for drawing conclusions. As a response to those problems, calls for actions have been raised, among others by the American Statistical Association (ASA), to rectify these issues, for instance, by modifying or even abandoning NHST. In this paper, we study the reaction of the community on these discussions. Specifically, we conduct a scientometric analysis of bibliographic records to investigate the publication behavior about the usage of NHST. We conduct a trend analysis for the general community, for specific subject areas and for individual journals. Furthermore, we conduct a change-point analysis to investigate if there are continued movements or actual changes. As a result, we find that for the general community NHST is more popular than ever, however, for particular subject-areas and journals there is a clear heterogeneity and no uniform publication behavior is observable.

## 1 Introduction

In statistics and machine learning many methods for analyzing data have been developed over the decades. One of the most prominent ones among those may be null hypothesis significance testing (NHST). Originating from an amalgamation of the works by Fisher, Neyman and Pearson dating back to the 1930s [1], [2], [3], NHST is enormously popular to this day and in many fields considered an authoritative method. Despite its popularity and widespread application, NHST is afflicted by numerous misconceptions and misuses [4], [5], [6], [7], [8], [9], [10], [11]. Even worse, NHST has been identified as one reason for the *replication crisis* [12], [13], [14], [15]. While in general a better education will certainly help to gain a greater level of understanding of NHST preventing some of the above mentioned issues [16], [17], [18] stronger calls for actions have been raised. These are ranging from an abandoning of NHST [19] to mild changes as the lowering of the significance level [20] and modifications situated in between [21]. Also the American Statistical Association (ASA) released a “Statement on Statistical Significance and P-values” [22], [23] to provide a policy statement on p-values and statistical significance.

Considering the non-trivial nature of the problems surrounding NHST, a question of interest is how did the community react to all these discussions and the statement of the ASA. The purpose of this paper is to conduct a scientometric study addressing this question. In general, scientometrics, sometimes also called infometrics [24], studies the scientific activity of scientific communities by using quantitative methods and its underlying goal is to gain insights into the structure, dynamics, and impact of scientific research [25], [26]. To achieve this goal, scientometrics studies are using large-scale bibliographic data, e.g., as documented by publications in journals.

In our study, we use publication data from the Web of Science (WoS) allowing to gather information on high-quality publications related to NHST. By querying the WoS, we are able to identify journal publications that utilize NHST in their investigations. Additionally, we utilize the subject areas of the papers, the journals in which they appeared, and the year of publication, to categorize the publications. These categorizations allow us to analyze the resulting time course data of publications and explore trends in publication behavior. Overall, our approach provides a comprehensive and systematic means of analyzing NHST-related publications, which can yield valuable insights into the field's evolution over time. In addition, we conduct a change-point analysis to investigate if there are continued trends or actual changes in the publication behavior. Furthermore, we extend our analysis to include proposed statistical alternatives to, or complements of, NHST, such as publications about confidence intervals and Bayes factors. To the best of our knowledge, no similar analysis has been conducted to study the trends of publications related to NHST in the scientific community. Hence, our research provides a unique perspective on the evolution of statistical methods in scientific research and can inform future research practices in this field.

This paper is organized as follows. In the next section, we present the data from the Web of Science (WoS) we use for our study and the methods for our numerical analysis. Then we present numerical results from the analysis and a detailed discussion of our main findings. The paper finishes with a brief summary and concluding remarks.

## 2 Methods

For our scientometric analysis, we use data from the Web of Science (WoS) citation and reference database. WoS is provided by Clarivate allowing a comprehensive access to publication records about all academic disciplines, including fields from science, engineering and humanities.

### 2.1 Publication data

We utilize the advanced search functionality of WoS allowing to specify search tags in combination with Boolean functions. This allows the creation of specific queries for obtaining selected search results. Specifically, to find publications about null hypothesis significance testing (NHST), we search for combinations of the following phrases: p-value, statistical significance, null hypothesis and hypothesis testing. That means we consider singular as well as plural forms of the search phrases and their logical “OR” combination. These terms have been chosen because when describing or reporting results about NHST some or all of these terms are frequently used. According to our results this allows to identify a large portion of relevant articles.

We distinguish between two different article types. The first is research articles and the second editorials. For editorials we include also review papers because these provide surveys and guidelines to the community rather than original research results. The time line we consider for all searches is from 2010-01-01 to 2022-12-31 whereas publications for each year are counted between January and December.

To study field-specific trends, we created subject-specific queries. Specifically, for the following, we combined results from different WoS categories.

• Medicine: medicine, general & internal, oncology, surgery, clinical neurology, public, environmental & occupational health, radiology or nuclear medicine & medical imaging.
• Psychology: psychology, psychology multidisciplinary or psychiatry)
• Economics: economics, business or management
• Pharmacology: pharmacology & pharmacy
• Biology: biology, biochemistry & molecular biology, cell biology, development biology, evolutionary biology, genetics & heredity, reproductive biology, marine & freshwater biology
• Engineering: engineering or physics
• Computer Science: computer science or mathematics
• Statistics: statistics & probability

These fields represent important areas where NHST is used frequently.

To obtain publication data for journals, we use the search functionality provided by the journals themselves. The advantage is that the journals allow a full text search not limited to title, abstract and keywords as WoS. Specifically, we obtain publication data from Nature, Nature Medicine, Nature Communications, Proceedings of the National Academy of Sciences (PNAS) and PLOS ONE. We use Nature as an interdisciplinary top tier journal, Nature Medicine because it is a top tier journal in medicine and Nature Communications, PNAS and PLOS ONE because these journals are not focused on single subjects but publish across all academic disciplines. Furthermore, by using three journals from one publisher (Nature, Nature Medicine, Nature Communications), we can study if there is an editorial policy on the top level that is passed down to the individual journals or if these journals operate with independent policies.

### 2.2 Analysis methods

In order to analyze the publication data, we use three different methods. The first two are based on linear regression [27] and the third one is a change point analysis [28].

#### 2.2.1 Regression analysis

The collected data sets of all categories consist of 13 data points corresponding to the years from 2010 to 2022. For the first type of regression analysis, we split the data sets into two parts, one containing the data points from 2010 to 2015 and the second from 2017 to 2022. For each part, we perform a separate, one-dimensional linear regression

(1) $$y\approx \beta_{1}x+\beta_{0}$$

and report the coefficient of the slope, $\beta_{1}$, and the corresponding p-value. We conduct this regression separately for the number of publications and proportions.

#### 2.2.2 Difference-point regression (DPR)

In addition, we perform a second type of regression analysis for the difference-points defined by

(2) $$y^{\prime }(i)=y(2017+i)-y(2010+i)$$

with i∈{0,…,5}. The means, we substract the observed values from different years which are eight years apart to obtain the difference-points $y^{\prime }(i)$. The resulting regression is conducted for the data points given by ${\left\{(i,y^{\prime }(i))\right\}}_{i=0}^{5}$. In order to distinguish this regression from the above one, we call it difference-point regression (DPR). Also the DPR, we conduct separately for the number of publications and proportions.

#### 2.2.3 Change-point analysis

For the change-point analysis, we use a Pettitt test [29]. The Pettitt test is a non-parametric rank-test based on the Mann-Whitney two sample test and its underlying idea is to test for a change of distribution in a sequence of observations. That means its Null and Alternative hypothesis for a two-sided test are given by:

• H_0_, Null hypothesis: There is no change of distribution in a sequence of observations.
• H_1_, Alternative hypothesis: There is a change of distribution in a sequence of observations.

For a sequence of random variables $X_{1},\ldots ,X_{T}$ there is a change-point at *τ* if all $X_{t}$ with t≤τ have a common distribution function $F_{1}$, and all $X_{t^{\prime }}$ with $t^{\prime }>\tau$ have a common distribution function $F_{2}$, with $F_{1}\neq F_{2}$.

The test statistic of Pettitt's test is given by

(3) $$K_{\tau }=\max_{t\in \{1,\ldots ,T\}}|U_{t,T}|$$

for

(4) $$U_{t,T}=2R_{t}-t(T+1)$$

(5) $$R_{t}=\sum_{i=1}^{k}r_{i}$$

where the $r_{i}$ with i∈{1,…,T} are the ranks of the sequence elements $X_{i}$. The possible change-point *τ* is located at the maximum of $\left|U_{t,T}\right|$, i.e., $K_{\tau }=U_{\tau ,T}$.

The p-value for a two-sided test can be approximated by

(6) $$\text{p-value}\approx 2\exp(\frac{-6K_{\tau }^{2}}{T^{3}-T^{2}}).$$

It is important to note the Pettitt's test does not assign p-values to all possible points but only to the most likely change-point given by Eqn. (3).

All parts of the analysis are conducted by using the statistical programming language R [30].

## 3 Results

In this section, we present the results of our analysis that is subdivided into four parts. First, we provide a global overview of all publications on the community-level about NHST. Second, we present a detailed analysis for different subject areas and, third, for journal publications. Forth, we study alternative statistical approaches for NHST on a global community-level and for different subject areas and journals. The fourth part of the results section provides information about a change-point analysis for all time course data.

### 3.1 Global overview

We start our analysis by providing a global overview of all publications about NHST. This will give us insights into publication behavior at the community-level, including all subject areas and journals. In the following sections, we will investigate details at the subject- and journal-levels.

**Figure 1:**
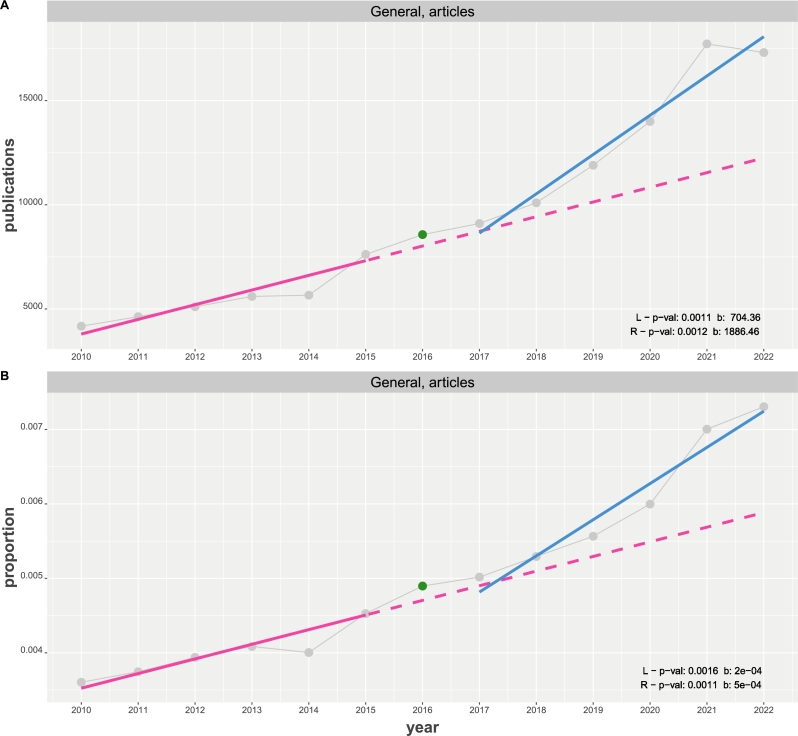
Time course of general publications of articles about NHST from 2010 to 2022. Results from two linear regressions (in red (L) and blue (R)) are shown in the bottom, right corner. A: The y-axis corresponds the number of publications. B: The y-axis corresponds the proportion of normalized publications.

Fig. 1 shows a time course of general publications of research articles about NHST from 2010 to 2022 collected from the Web of Science (WoS). That means the considered publications are the accumulation of all research articles listed by WoS irrespective of the nature of the field or journal. The difference between the two figures is that Fig. 1 A shows the number of publications whereas Fig. 1 B gives the proportion of normalized publications where a proportion is obtained by

(7) $$\text{proportion }|\text{ year}=\frac{\text{number of publications about NHST | year}}{\text{number of all publications | year}}.$$

Here the denominator corresponds to all research articles published in a given year as listed by WoS. This normalization provides an adjustment for a changing number of publications per year and allows a relative comparison instead of an absolute one. Fig. 1 A and B show the number of publications and their proportions using gray points, with gray lines connecting consecutive data points.

In order to explore possible changes in the publication behavior of the general community as a possible consequence of the ASA article [23] in 2016, highlighted in green in Fig. 1, we fit two regression lines. One regression line from 2010 to 2015 and one from 2017 to 2022. Fig. 1 A and B show the first regression on the left-hand side (L) in red and the second on the right-hand side (R) in blue. To make a comparison between both lines easier, we extend the first regression to 2022 shown as dashed line.

**Figure 2:**
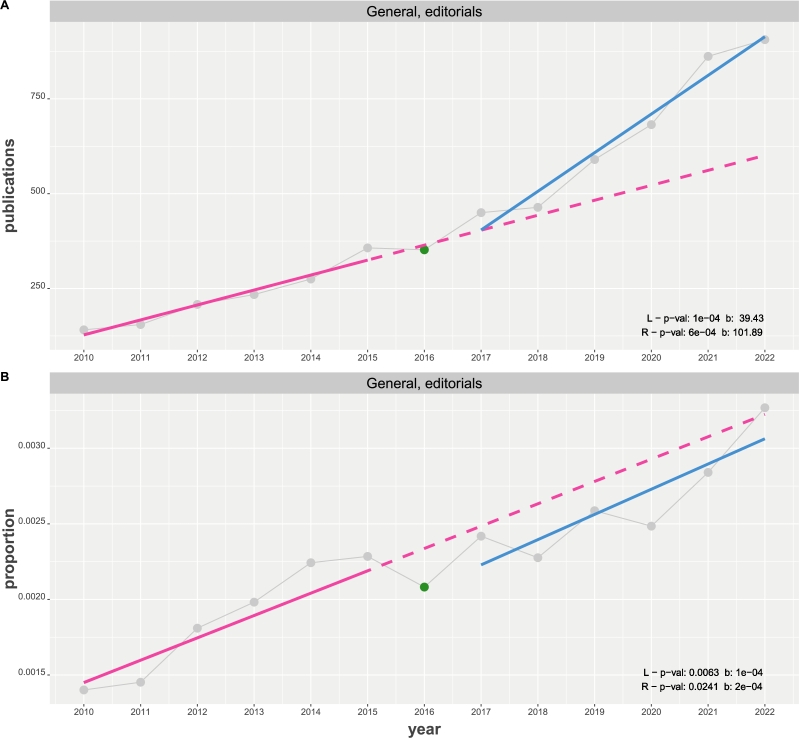
Time course of general publications of editorials about NHST from 2010 to 2022. Results from two linear regressions (in red (L) and blue (R)) are shown in the bottom, right corner. A: The y-axis corresponds the number of publications. B: The y-axis corresponds the proportion of normalized publications.

**Figure 3:**
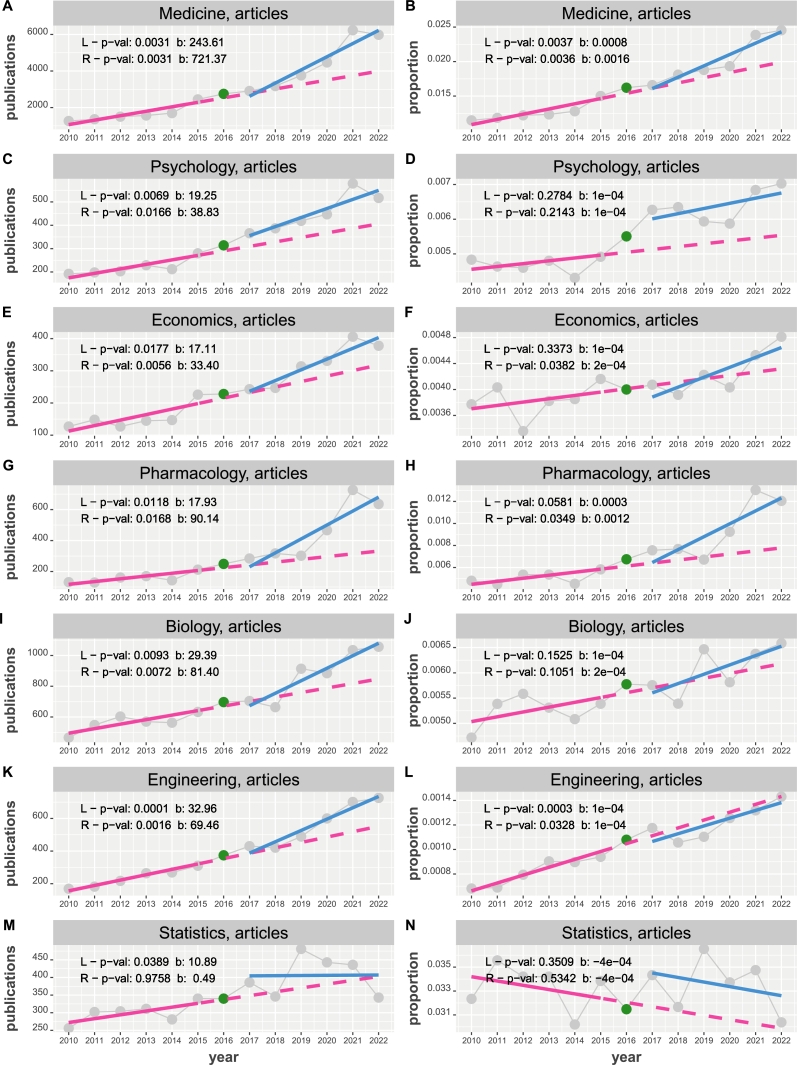
Time course of subject-specific publications of articles about NHST from 2010 to 2022. Results from two linear regressions (in red (L) and blue (R)) are shown in the top left corner. Left column: The y-axis corresponds the number of publications. Right column: The y-axis corresponds the proportion of normalized publications.

**Figure 4:**
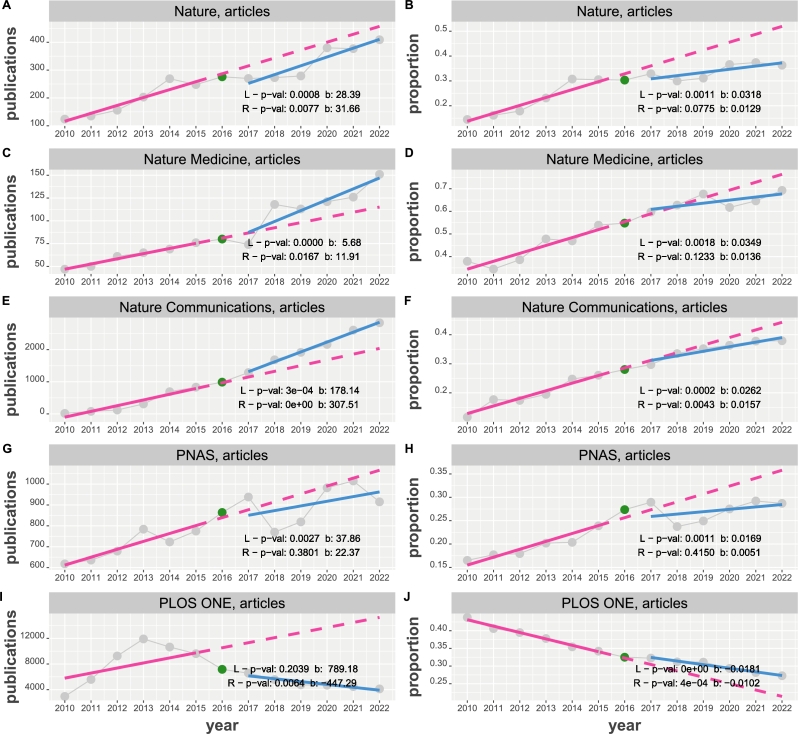
Time course of articles about NHST published in the journals Nature, Nature Medicine, Nature Communications, PNAS and PLOS ONE from 2010 to 2022. Results from two linear regressions (in red (L) and blue (R)) are shown in the top, left corner. Left column: The y-axis corresponds the number of publications. Right column: The y-axis corresponds the proportion of normalized publications.

**Figure 5:**
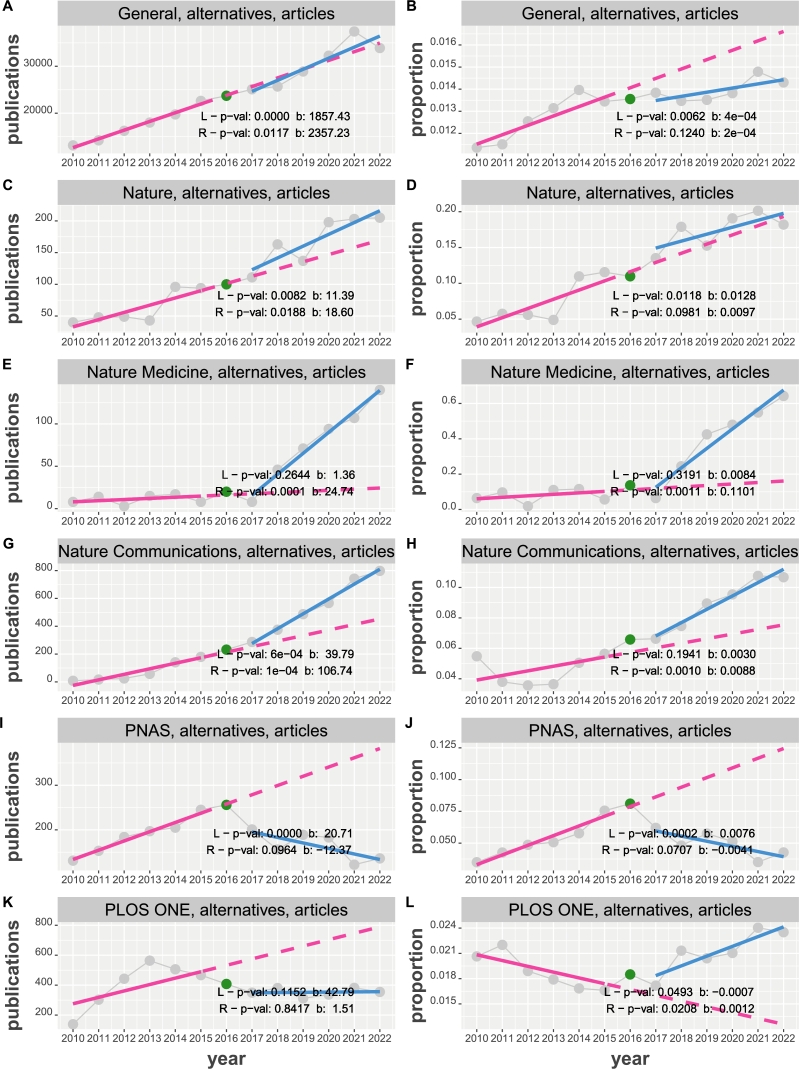
Time course of general and journal publications of research articles about alternative statistical approaches from 2010 to 2022. Results from two linear regressions (in red (L) and blue (R)) are shown in the top, left corner. A: The y-axis corresponds the number of publications. B: The y-axis corresponds the proportion of normalized publications.

From the two linear regression models, one can see that all p-values for the slopes (corresponding to $\beta_{1}$; see Eqn. (1)) are significant for a significance level of α=0.05 with a Bonferroni correction [31] with all slopes are positive. Here (and in subsequent Figure 2, Figure 3, Figure 4, Figure 5) a conservative Bonferroni correction considers all tests in a subfigure since these are the results compared with each other. Furthermore, one can see that the slope of right linear regression is more than twice as large as the slope of the left regression line. This holds for the number of publications as well as the proportions. As a quantification for this observation, we estimate the slope factor, given by $SF=\beta_{1}(R)/\beta_{1}(L)$, and obtain SF=2.67 for the number of publications and SF=2.50 for the proportions.

Fig. 2 A, B presents a similar analysis as in Fig. 1, but for editorials instead of research articles. Also here we do not select publications of particular fields or journals but consider all editorials published by the general community as listed by WoS. While, numerically, the results of the linear regression models are similar to the ones for the research articles shown in Fig. 1 (tests for all slopes are significant) the visual impression of the results for the proportions is different. Specifically, the values of the proportions are decreased below the baseline regression in red. That means relative to all published editorials, the editorial publications (proportions) about NHST are on a lower level than they would be if the trend from 2010 to 2015 would have continued. For the slope factors we find SF=2.58 for the number of publications and SF=2.00 for the proportions.

### 3.2 Subject-specific evolution

Our next study extends the above analysis to subject-specific publications. For this, we search the WoS for publications in the seven areas; medicine, psychology, economics, pharmacology, biology, engineering and statistics - as defined in Section 2). This will provide insights into the usage of NHST in these fields.

Fig. 3 shows the results of this analysis. On the left column the y-axis corresponds the number of publications and on the right column the y-axis gives the proportion of normalized publications. First, we would like to note that 15 out of the 28 tests about the significance of the regression slopes are statistically significant whereas 13 are not. Interestingly, 11 of the non-significant results are for proportions and only 2 are for the number of publications. Specifically, in Fig. 3, the non-significant results are for proportions in psychology (both), economics (both), pharmacology (both), biology (both), engineering (R), statistics (both) and the number of publications in statistics (both).

The slope factors β(R)/β(L) for the number of publications are 2.96 (medicine), 2.01 psychology, 1.95 (economics), 5.02 (pharmacology), 2.76 (biology), 2.10 (engineering) and 0.04 (statistics) and for the number of proportions we find: 2.00 (medicine), 1.00 psychology, 2.00 (economics), 4.00 (pharmacology), 2.00 (biology), 1.00 (engineering) and 1.00 (statistics). Interestingly, there is not one field that shows a significant decline in either the number of publications or proportions but psychology, economics, pharmacology, biology, engineering and statistics show a sideward movement for the proportions.

### 3.3 Journal-specific evolution

For our next analysis, we study journal-specific publications of articles about NHST. For this study, we select five well-established journals; Nature, Nature Medicine, Nature Communications, PNAS and PLOS ONE. We selected four of these because they represent broad, interdisciplinary journals whereas Nature Medicine is focused on medical problems which are among the most important applications of NHST.

The results of this analysis are shown in Fig. 4. Out of the 20 tests for the slopes of the linear regression, we find 5 that are non-significant. Specifically, for the proportions these are obtained for Nature (R), Nature Medicine (R) and PNAS (R) whereas for the number of publications these are for PNAS (R) and PLOS ONE (L). It is interesting to highlight the results for PLOS ONE because it is the only journal that has significant slopes with a negative sign (for the number of publications and proportions).

For the factors of slopes β(R)/β(L) where both regressions are significant, we find for the number of publications 1.11 (Nature), 2.09 (Nature Medicine) and 1.73 (Nature Communications) and for the proportions 0.59 (Nature Communications) and 0.56 (PLOS ONE). We would like to highlight that only the factors for the proportions are less than one hinting a decrease in a trend.

### 3.4 Alternative statistical approaches

As an alternative for NHST several approaches have been suggested, maybe most prominently confidence intervals and Bayes factors. In the following, we repeat a similar analysis as before but by searching for these alternative approaches.

Fig. 5 A and B, shows results for general publications of research articles about alternative statistical approaches to NHST. Interestingly, only the right regression slope (R) of the proportions is not significant indicating a sideward movement of the normalized publications.

Similar results for journal publications (Nature, Nature Medicine, Nature Communications, PNAS and PLOS ONE) of research articles about alternative statistical approaches are shown in Fig. 5 C - L. Out of 20 tests for the slopes, 9 are not significant. For the number of publications the non-significant results are for Nature Medicine (L), PNAS (R) and PLOS ONE (both) and for proportions these are for Nature (R), Nature Medicine (L), Nature Communications (L), PNAS (R) and PLOS One (L).

For the factors of slopes β(R)/β(L) of the proportions we find 0.75 (Nature), 13.10 (Nature Medicine), 2.93 (Nature Communications), -0.53 (PNAS) and 1.71 (PLOS ONE).

### 3.5 Change-point analysis

So far, we conducted our analysis around the year 2016 to see if there are differences in publication behavior for articles about NHST before and after this year. Now, we remove this focal point and refrain from making any assumptions about a specific year when the publication behavior of the community might change. Instead, we conduct a change-point analysis that allows to identify the year of such a potential change [32], [33]. Specifically, for a sequence of random variables $X_{1},\ldots ,X_{T}$ a change-point at *τ* separates the sequence into two parts, $X_{1},\ldots ,X_{\tau }$ and $X_{\tau +1},\ldots ,X_{T}$, i.e., it is the last year before the series actually changes and not the first year of the second time series. Statistically, the random variables $X_{t}$ on the left-hand side (L), i.e., t≤τ and the random variables on the right-hand side (R), i.e., t>τ, have a common but different distribution function with $F_{L}\neq F_{R}$.

For identifying a possible change-point, *τ*, in the time course data of the publications, we use Pettitt's test [29]. The results of this analysis are shown in Table 1 where we use a significance level of α=0.05 with a Bonferroni correction. In this table, the first column shows the figure ID of our results. That means, we conduct a change-point analysis for all time course data studied so far. To simplify the distinction between significant and non-significant results, we place non-significant p-values in brackets.

**Table 1:** Change-point analysis of time course data of publications using a Pettitt test. The first column shows the figure ID of the preceding results, column two shows the potential change-point and column three its corresponding p-value. Non-significant results are placed in brackets.

Figure ID	Change point	p-value	Change size
1 A: general articles	6	0.0228	6341
1 B: general articles	6	0.0228	0.001594301
2 A: general editorials	7	0.0228	394.5
2 B: general editorials	7	(0.0346)	0.0007168738
3 A: Medicine articles	6	0.0228	2127
3 B: Medicine articles	6	0.0228	0.006488235
3 C: Psychology articles	6	0.0228	201
3 D: Psychology articles	6	0.0228	0.001529822
3 E: Economics articles	6	0.0228	159
3 F: Economics articles	5	(0.0748)	0.0003561216
3 G: Pharmacology articles	6	0.0228	173.5
3 H: Pharmacology articles	6	0.0228	0.003059051
3 I: Biology articles	6	0.0228	296.5
3 J: Biology articles	6	(0.0346)	0.0007608856
3 K: Engineering articles	6	0.0228	266.5
3 L: Engineering articles	6	0.0228	0.0003801214
3 M: Statistics articles	7	0.0228	99.5
3 N: Statistics articles	4	(1.0000)	-0.0008428636
4 A: Nature articles	6	0.0228	136.5
4 B: Nature articles	4	(0.0748)	0.147556
4 C: Nature Medicine articles	5	(0.0346)	52.5
4 D: Nature Medicine articles	6	0.0228	0.2051883
4 E: Nature Communications articles	6	0.0228	1604.5
4 F: Nature Communications articles	6	0.0228	0.1486307
4 G: PNAS articles	6	(0.0514)	199.5
4 H: PNAS articles	5	(0.0346)	0.08478454
4 I: PLOS ONE articles	6	(0.2040)	-3367.5
4 J: PLOS ONE articles	4	0.0228	-0.08239816
5 A: General alternatives articles	6	0.0228	11715.5
5 B: General alternatives articles	6	(0.0748)	0.00195307
5 C: Nature alternatives articles	6	0.0228	103
5 D: Nature alternatives articles	7	0.0228	0.09158669
5 E: Nature Medicine alternatives articles	8	(0.0346)	83
5 F: Nature Medicine alternatives articles	8	(0.0346)	0.408947
5 G: Nature Communications alternatives articles	6	0.0228	406.5
5 H: Nature Communications alternatives articles	6	0.0228	0.03901977
5 I: PNAS alternatives articles	8	(0.4640)	-43
5 J: PNAS alternatives articles	3	(0.5860)	0.01324403
5 K: PLOS ONE alternatives articles	2	(0.5860)	200.5
5 L: PLOS ONE alternatives articles	8	(0.1490)	0.003495726

From Table 1, one finds that 24 (corresponding to 60%) out of the conducted 40 tests are significant which means that the corresponding 24 time course data potentially contain a change-point. Specifically, the observed frequencies of significant change-points are 1(4),20(6)and3(7) corresponding to the years 2014 (4), 2016 (6) and 2017 (7). It is interesting to note that the most frequent change-point occurs at τ=6 which is 2016 corresponding to 83.3% of all significant results. Overall, these results indicate that in most cases, there is a notable transition between two time intervals of variable lengths, revealing a non-steady publication behavior. This pattern is observed at all levels of our analysis: community-level, subject-level, and journal-level.

## 4 Discussion

Null hypothesis significance testing (NHST) is truly a method that finds interdisciplinary applications in all fields of science. In addition, it has a gold-standard reputation in some fields, e.g., medicine and psychology when it comes to the selection of analysis methods and so for many decades [34], [35]. Maybe similar far back are dating criticisms that flared-up considerably with the debate about the reproducibility crisis in recent years [36]. In this paper, we do not provide additional pros or cons for using NHST, nor do we assume a particular opinion about this method. Instead, we study the publication behavior of the community at different levels.

While criticisms of NHST are ongoing since many years, the statement by the American Statistical Association (ASA) in 2016 based on the views of a group of over 20 experts can be seen as the culmination of this phase [22]. For this reason, in the first part of our analysis, we use the year 2016 as the center-point to identify changes in publication behavior before and after this year. In contrast, in the second part of our analysis, we do not single out any particular year but use change-point analysis to identify such points from the time course data itself. Furthermore, to obtain detailed insights, we group publications into well-defined categories, allowing the exploration of transitions and trends in the general community, specific fields, or journals. In the following sections, we compartmentalize our findings into three main parts, elaborating on the outcomes for (I) the general community, (II) publications specific to subjects and journals, and (III) alternative statistical approaches.

(I) Our results for the publication behavior of the general community show that the number of research articles about NHST are stronger increasing since 2016 than before. This holds for the observed absolute number of publications as well as the proportions, see Fig. 1 A and B, and is exemplified by slope factors of SF=2.76 and SF=2.50 for the number of publications and proportions respectively. For these time course data, even a change-point analysis identifies the start of a trend at 2016, see Table 1. This justifies in retrospective using the year 2016 as dividing point for the separation of the two regression models. To test if also the change in both regression models is significant, one from 2010-2015 and the other from 2017-2022, we perform an additional comparison by means of a regression analysis for the difference-points (DP) defined by

(8) $$y^{\prime }(i)=y(2017+i)-y(2010+i)$$

with i∈{0,…,5}. The means, we substract the observed values (either for the number of publications or proportions) from different years which are eight years apart to obtain the difference-points $y^{\prime }(i)$; see the Methods section for details about the difference-point regression (DPR). As a result from this regression analysis, we find a significant positive slope for the number of publications (p-value of 0.0171) and proportions (p-value of 0.0128). Hence, also the differences in the regression models for the number of publications and the proportions from 2010-2015 and from 2017-2022 are statistically significant.

Similarly, also the number of published editorials are increasing but the values of the proportions are lowered below the baseline regression continuing the trend from 2010 to 2015; see Fig. 2 B. That means, relative to all published editorials, editorials about NHST are still increasing but on a lower acceleration than before. Interestingly, the change-points for the editorials occur one year later than for the research articles at 2017; see Table 1. From these findings, one can see that especially publications of research papers of all subjects and journals are sill heavily utilizing NHST in studies across all communities and that there is an even stronger acceleration after 2016 compared to the years up to 2016.

(II) For subject-specific publications about NHST, we find also for medicine, pharmacology and engineering an increasing number of publications and proportions with a change-point at 2015. While this is also true for economics for the number of publications, for the proportions no change-point can be identified. For the difference-point regression, we find p-values of 0.021,0.0466,0.01000 (medicine, pharmacology and engineering) for the number of publications and 0.0208,0.0963,0.79349 for the proportions that means only for medicine both difference-point regressions are significant. This is important to note because it means that in the medicine community NHST is even more abandonedly used than before 2016 but not in the other fields.

Yet a different situation is observed for psychology, and biology. For these subjects, we find slope factors of SF=2.02 (psychology) and SF=2.77 (biology) for the number of publications and a change-point at 2015. Interestingly, for psychology and biology, none of the regression models are significant for the proportions. For the difference-point regression, we find p-values of 0.156,0.0384 (psychology, and biology) for the number of publications and 0.2572,0.345 for the proportions. That means only for the number of publications in biology there is a significant positive slope of the difference regression. Interestingly, a change-point is still detected for both fields at 2015. This indicates that for the psychology and biology community no accelerated increase occurs as measured by the proportions. Finally, statistics is different to all other fields because it is the only subject having a negative slope for the proportions; see Fig. 3 N, although non-significant. Overall, it seems fair to say that psychology, biology and statistics make a sideways movement for the proportions which is neither clearly increasing nor decreasing. This is interesting because it means on a subject-level not all fields behave uniformly but publications in some journals increase, while others do not. Importantly, none of the journals showed a decreasing trend in publications or proportions.

For journal-specific publications about NHST, we observe a similar diversity as for the subject areas. An example for a journal that shows an increase in the number of publications and proportions is Nature Communications with slope factors of SF=1.73 and SF=0.59, where the factor below one indicates a reduced increase compared to the baseline or a cooling off. Interestingly, also for Nature Communications, we identify a change-point at 2016. In contrast, while the number of publications in Nature and Nature Medicine also increase, the proportions of the journals are making a sideways movement. Yet a quite different behavior is displayed by PLOS ONE which is the only journal with a negative significant slope for the number of publications and proportions; see Fig. 4 I and J. Interestingly, for the number of publications no change point can be identified whereas for the proportions the change-point is at 2014. Hence, also for journal-specific publications about NHST, we observe a heterogeneous publication behavior that is not uniform across all journals. For reasons of clarity, we summarize our key findings in Table 2.

**Table 2:** Summary of key results. The first column indicates main levels and the second its category. The remaining columns show results for the proportions. Transition refers to the change from the interval 2010-2015 to the interval 2017-2022 whereas trend indicates the behavior after the change year.

Level	Category	Slope factor	Change year	Transition	Trend
community	articles	2.50	2016	increase	↑
	editorials	2.00	2017	steady increase	↑

subject	medicine	2.00	2016	increase	↑
	psychology	1.00	2016	steady	→
	biology	2.00	2016	steady	→
	engineering	1.00	2016	steady increase	↑
	statistics	1.00	-	steady	→ (neg slope)

journal	nature	0.41	2014	decrease	→
	nature medicine	0.39	2016	decrease	→
	nature com	0.59	2016	steady increase	↑
	PNAS	0.30	2015	decrease	→
	Plos One	0.56	2014	steady decrease	↓

(III) Finally, we conducted an analysis investigating alternative statistical approaches to NHST. First, we would like to note that to date there is no official replacement of NHST that would be generally accepted as such. This makes it difficult to specify a literature search because without a clear naming of such approaches they cannot be identified unambiguously. The following two examples from the literature demonstrate this problem [20] and [19]. While [20] suggests only a change in the value of the significance level setting it to 0.005 instead of 0.05, the latter article suggest to abandon the thresholding of the p-value and to replace it by a thorough discussion of all evidence available. Technically, the suggestion by [20] still utilizes formally NHST and is no actual alternative because the value of the significance level is anyway a parameter that can be decided by the analyst. In contrast, the suggestion by [19] differs significantly from NHST, but the proposed alternative is more of a process than a single, uniquely identifiable method. As a result, many publications that discuss “alternative” approaches—however they are defined—could (and likely will) also include NHST. On the other hand, excluding publications containing also NHST in addition to some other approaches would lose many (or all) alternatives suggested in [19]. Keeping these issues in mind, what we term ‘alternative approaches’ is much less well-defined and precise compared to NHST, which often includes studies involving confidence intervals and Bayes factors, sometimes in combination with NHST within the same paper.

From our numerical results regarding the publication behavior of the general community of alternative statistical approaches, we find an increase in the number of publications but a sideways movement for the proportions; see Fig. 5 A and B. Also, no change point could be detected for the proportions. Contrasting these results with the proportions for publications about NHST (Fig. 1 B) one can conclude that the usage of NHST is increasing compared to its alternatives and even more so since 2016. The difference-point regression (see Eqn. (8)) gives a non-significant positive slope (p-value of 0.792) for the number of publications and a negative significant slope (p-value of 0.03110) for the proportions. The latter confirms the impression given by Fig. 5 B that there is a cooling off after 2016.

For journal-specific publications about alternative approaches, we find an increase in the proportions for Nature Medicine and Nature Communications with a change-point at 2018 and 2016 respectively. Maybe the most curious behavior is observed for PLOS ONE that shows a significant negative slope up to 2015 and a significant positive slope from 2017 onward, however, without detectable change-point.

### 4.1 Additional comments

For our exploratory analysis, we used a number of different methods, including linear regression, statistical testing, difference-point regression and change-point analysis. Regression models, statistical testing, and other statistical approaches applied to text features have a long history in text mining, information retrieval, and natural language processing across various application domains [37], [38], [39], [40], [41], [42]. However, there is no off-the-shelf method for trend analysis based on text data that addresses our specific research question. For this reason, we combined several approaches to uncover this information. Because this is a procedural approach rather than a monolithic method, we refer to it as exploratory analysis, in the spirit of Tukey [43], indicating that more refined methods may exist that could yield similar results more elegantly. This would be interesting to study in the future.

It is interesting to note that our analysis has similarities to [19] suggesting to gather and evaluate all evidence available, including p-values without application of a threshold (or significance level), instead of basing an analysis on NHST. However, a necessary consequence thereof is the exploratory character of our analysis, as mentioned above.

When preparing these articles, we tried also to find advocates for NHST. However, the only (partial) defender we could find is [44]. In light of this overwhelming negative publicity of NHST, the results from our study may surprise because they represent the publication behavior of the general community. If this behavior is an example for or against the wisdom of the crowds is at this point unclear. In general, the “wisdom of the crowds” [45] proposed that large groups of people or crowds can be collectively smarter than individuals. This implies that the collective knowledge and opinions of a group are better at decision-making than an individual. At this stage, it seems worth to go back to re-address some previous points in discussing NHST, including the detailed specification of alternative approaches. These should be as simple to execute as a NHST in order to avoid confusion. Furthermore, there may be a need for a more fundamental discussion regarding inductive inference.

Lastly, we would like to remark that when using NHST in studies, it often requires complementing with multiple correction methods [46]. Among the most popular methods are those by Bonferroni and Benjamini & Hochberg [47], but many others exist [48]. Regarding our research question, we do not differentiate between publications using NHST with or without multiple correction methods, as the application of NHST itself is our primary focus. However, whenever NHST is used practically, the need for multiple correction methods should be evaluated, and appropriate approaches should be chosen.

## 5 Conclusion

There are few methods in statistics that have a similar formidable status as null hypothesis significance testing (NHST). Still, there are undeniable problems with NHST because of misuses and misconceptions that can be found extensively in the scholarly literature no least in the context of irreproducible studies. Aside from the continued calls for actions over the years to rectify these issues, e.g., by modifying or even abandoning NHST, the statement by the American Statistical Association (ASA) in 2016 marked a distinguished event. Utilizing publication records from the Web of Science (WoS), we conducted a scientometric analysis investigating the publication behavior of the community about NHST to see if there are detectable differences due to those discussions.

In summary, for the general community our findings indicate that NHST is more popular than ever. However, on the subject- and journal-level there is a clear heterogeneity and no uniform publication behavior is observable. It is always difficult to predict in which direction a field will develop but based on our findings it seems fair to say that there are no indications that NHST will be abandoned any time soon. Instead, it's popularity for the general community is still increasing. Yet, at the same time there are subjects like psychology, biology and statistics in which the popularity of NHST does neither clearly increase nor decrease but maintains a similar status. It will be interesting to see if and how there will be further concerted events such as the ASA statement to educate the general community about NHST and if those measures lead to a different outcome.

## CRediT authorship contribution statement

**Frank Emmert-Streib:** Writing – review & editing, Writing – original draft, Methodology, Conceptualization.

## Declaration of Competing Interest

The authors declare that they have no known competing financial interests or personal relationships that could have appeared to influence the work reported in this paper.

## Data Availability

Data will be made available on request.
